# GOLPH3 Promotes Cancer Growth by Interacting With STIP1 and Regulating Telomerase Activity in Pancreatic Ductal Adenocarcinoma

**DOI:** 10.3389/fonc.2020.575358

**Published:** 2020-10-02

**Authors:** Kebing Wang, Shuai Jiang, Anpei Huang, Ying Gao, Baogang Peng, Zhi Li, Wenbin Ma, Zhou Songyang, Shihong Zhang, Meifang He, Wen Li

**Affiliations:** ^1^Laboratory of General Surgery, The First Affiliated Hospital, Sun Yat-sen University, Guangzhou, China; ^2^Key Laboratory of Gene Engineering of the Ministry of Education and State Key Laboratory of Oncology in South China, School of Life Sciences, Sun Yat-sen University, Guangzhou, China; ^3^Department of Hepatobiliary Surgery, The First Affiliated Hospital, Sun Yat-sen University, Guangzhou, China; ^4^Department of Pathology, The First Affiliated Hospital, Sun Yat-sen University, Guangzhou, China

**Keywords:** GOLPH3, STIP1, hTERT, cell proliferation, pancreatic ductal adenocarcinoma (PDAC)

## Abstract

Overexpression of Golgi phosphoprotein 3 (GOLPH3) predicts poor prognosis and is a potential therapeutic target in pancreatic ductal adenocarcinoma (PDAC). However, its role and underlying molecular mechanisms in the progression of PDAC remain unknown. In the present study, using high-throughput bimolecular fluorescence complementation (BiFC) analysis, we identified that stress-inducible protein-1 (STIP1) interacts with GOLPH3 and confirmed the interaction using co-localization and co-immunoprecipitation. The levels of GOLPH3 and STIP1 in PDAC tissues and adjacent non-cancerous pancreatic tissues were determined using immunohistochemistry (IHC) and quantitative real-time reverse transcription PCR. Real-time Quantitative-telomere repeat amplification (Q-TRAP) was applied to detect relative telomerase activity, and cell proliferation was measured when small interfering RNAs targeting *GOLPH3* or *STIP1* were transfected into PDAC cell lines. BALB/c nude mice were used to assess tumor growth inhibition of BXPC3 cells stably transfected with *GOLPH3* short hairpin RNA. In summary, GOLPH3 was found to interact with STIP1 and both proteins were overexpressed and co-localized in PDAC tissues and cell lines. Moreover, suppression of *GOLPH3* expression using shRNAs in PANC1 and BXPC3 cells inhibited tumor cell proliferation both *in vitro* and *in vivo*. Mechanistically, GOLPH3 interacts with STIP1 to activate telomerase reverse transcriptase (hTERT) and telomerase activity by c-Myc, and then upregulates cell cycle-related signaling proteins, including cyclin D1, to promote tumor cell growth, suggesting that disrupting the interaction between STIP1 and GOLPH3 would be a promising new strategy to treat PDAC.

## Introduction

Pancreatic ductal adenocarcinoma (PDAC) is a highly lethal malignancy with poor prognosis, and is the fourth leading cause of cancer-related death in the USA (1) and the seventh in China (2). A recent study showed that the overall survival (OS) of pancreatic cancer is very poor, with a median survival time (MST) of 7.8 months, and 1- and 5-year survival rates of 35.0 and 4.4% respectively, in Shanghai, China (3). Although some advances have been made in PDAC therapy, the long-term survival and prognosis for patients with PDAC is not satisfactory. To date, the only curative treatment available for PDAC is surgical resection. However, <20% of patients have surgically resectable tumors at the time of diagnosis because the majority of patients are not diagnosed until their disease is in the late stage (4–6). Therefore, determining the underlying mechanism of tumor growth is urgently needed for early diagnosis and effective treatment.

Golgi phosphoprotein 3 (GOLPH3), also known as GPP34, GMx33, MIDAS, or yeast Vps74p, is a highly conserved cytosolic trans-Golgi-associated protein present in yeast to humans, with a molecular weight of 34 kDa (7, 8). GOLPH3 is a phosphatidylinositol-4-phosphate (PI4P) binding protein in its proper Golgi localization and mutant forms of mammalian GOLPH3 (R90L and R171A/R174L) that do not bind PI4P failed to localize to the Golgi (9). GOLPH3 mediates several cellular functions, such as regulation of the Golgi architecture and cytokinesis, and the response to DNA damage (10). Dippold et al. reported that GOLPH3 interacts with Myosin 18A (MYO18A) to bind F-actin to maintain Golgi morphology and vesicular trafficking from the Golgi to plasma membrane (9). *GOLPH3* was the first reported oncogene whose encoded protein is localized to the Golgi and is frequently amplified in various solid tumors (11). Overexpression of GOLPH3 promotes the development and progression of several tumors, including breast cancer (12), colon cancer (13), gastric cancer (14), renal cancer (15), and epithelial ovarian cancer (16). Our previous study demonstrated that overexpression of GOLPH3 predicates poor prognosis and clinical progression in PDAC (17). The oncogenic activity of GOLPH3 is primarily related to the activation of several signal transduction pathways, such as the protein kinase B (AKT)/mechanistic target of rapamycin (mTOR) pathway (11), the Wnt/β-catenin pathway (15), and the nuclear factor kappa B (NF-κB) pathway (18). Several proteins interact with GOLPH3 to regulate downstream targets for tumor progression. Scott et al. proposed that *GOLPH3*, as a target of 5p13 amplification, could serve as a potent proto-oncogene in human cancers (11, 19), and found that overexpression of GOLPH3 could function with the retromer subunit VPS35, *via* the AKT/mTOR pathway, to promote cancer cell proliferation (11). Tumors that overexpress GOLPH3 are more sensitive to rapamycin treatment, indicating that the GOLPH3 level might be a positive predictor of rapamycin sensitivity. GOLPH3 suppresses the forkhead box O1 (FOXO1) transcription factor *via* AKT signaling to regulate the expression of cell-cycle inhibitors, thereby contributing to cell proliferation and tumorigenesis in breast cancer (20). Data from our research group supported a role for GOLPH3 in PDAC. We demonstrated that GOLPH3 levels correlate closely with PDAC progression, thus GOLPH3 might be a novel target for PDAC therapy (17). However, the biological interactions between GOLPH3 and other proteins as well as the regulation of molecular pathways and key events related to tumorigenesis in PDAC are unclear. Studying protein-protein interactions (PPIs) is critical to our understanding of signaling pathways. Therefore, based on PPIs, the present study aimed to identify proteins that interact with GOLPH3 and explore the biological mechanism of the interaction to gain a deeper understanding of the relationship between GOLPH3 and PDAC carcinogenesis.

To gain insights into the molecular mechanisms of GOLPH3's biological functions, we identified potential GOLPH3 interaction partners using a high-throughput bimolecular fluorescence complementation (BiFC) assay, a method that can visualize protein interactions in living cells (21). This method is based on the discovery that two non-fluorescent fragments of a fluorescent protein can form a fluorescent entity when in close proximity (22). The BiFC strategy has been utilized for a variety of applications, including the visualization of protein interactions (21), determination of subcellular localizations (23), and investigation of biological functions of PPIs (24). In the present study, a yellow fluorescent protein (YFP) N terminal fragment fused to GOLPH3 and a YFP C terminal fragment fused to an ORFeome library contained approximately 18,000 human open reading frames (ORFs) were cotransfected into HTC75 cells, and then the BiFC of YFP was detected to identify interactions of GOLPH3 with these proteins. These experiments revealed that stress-inducible protein-1 (STIP1) is a novel GOLPH3 binding partner. Furthermore, we found that the GOLPH3-STIP1 interaction was mechanistically associated with the promotion of telomerase reverse transcriptase (hTERT) as well as telomerase activity by c-Myc, which subsequently upregulated cyclin D1 to promote tumor cell growth. Our results showed that GOLPH3, acting as an oncoprotein, interacted with STIP1 to regulate telomerase activity and promote tumor progression, which might provide a new therapeutic target for PDAC.

## Materials and Methods

### Bimolecular Fluorescence Complementation (BiFC) Screening

HTC75 or HEK293T cells were cultured in Dulbecco's Modified Eagle's Medium (DMEM, Gibco, Gaithersburg, MD, USA) containing 10% fetal bovine serum (FBS) (Gibco) and 1% streptomycin/penicillin (Gibco) at 37 °C and 5% CO_2_. For BiFC, YFP is divided into two parts that cannot emit fluorescence even when both YFP parts are co-expressed in mammalian cells. However, when YFP fragments are fused with two proteins that naturally associate, the interaction between these proteins brings YFPn (amino terminus of YFP, residues 1–155) and YFPc (carboxy terminus of YFP, residues 156–239) close enough to reform a functional YFP protein. Proteins tagged with YFPn and YFPc, respectively, were stably co-expressed in HTC75 cells for flow cytometry analysis. For GOLPH3-BiFC screening, BiFC positive cells were sorted for four rounds to reach a positive rate more than 90%. cDNA was produced using cells sorted from the final sorting. The ORF sequence from the cDNA was amplified by PCR using BiFC vector specific primers, purified using a PCR clean-up kit (Qiagen, Shanghai, China), and subjected to high-through-put sequencing.

### Patients and Clinical Samples

Surgically resected PDAC tissues and adjacent non-cancerous pancreatic tissues were obtained from patients with PDAC who had undergone surgical resection in the Department of Hepatobiliary Surgery, the First Affiliated Hospital of Sun Yat-sen University (Guangzhou, China). The studies involving human participants were reviewed and approved by the Institutional Ethical Review Boards of the First Affiliated Hospital of Sun Yat-sen University (Ethical code number: 201515) and informed consent was obtained from the patients for the use of these clinical materials in this study.

### Immunoprecipitation (IP) Assay

Green fluorescent protein (GFP)/VPS35/STIP1 ORF sequences were cloned into the CMV promoter-driven Flag tagged pBabe-based vector for transient expression. The Flag-tagged construct was transiently transfected into HEK293T cells. At 48 h after transfection, the cell pellet was harvested and lysed in NETN buffer (1 M Tris-HCl pH 8.0, 1 mM EDTA, 100 mM NaCl, 0.5% NP-40, 1mM DTT and proteinase inhibitor cocktail (Sigma, St. Louis, MO, USA). The proteins in the cell lysate were immunoprecipitated using anti-Flag affinity agarose beads. The agarose beads were washed in NETN buffer four times. For western blotting, cell lysate or the IP-elution was boiled with western blotting loading buffer for 10 min before SDS-PAGE and antibody probing. The antibodies used were rabbit polyclonal anti-GOLPH3 (Abcam, Cambridge, MA, USA; ab236296), rabbit polyclonal anti-flag (Sigma, F7425), and anti-flag M2 Affinity Gel (Sigma, A2220).

### Western Blotting

PANC1 and BXPC3 cells and six pairs of PDAC tumor specimens and adjacent non-cancerous specimens were obtained and lysed in protein lysis buffer, comprising 50 mM Tris (pH, 7.5), 100 mM NaCl, 1 mM EDTA, 0.5% NP40, 0.5% Triton X-100, 2.5 mM sodium orthovanadate, 10 μM protease inhibitor cocktail, and 1 mM phenylmethylsulfonyl fluoride. Protein were separated by 10% SDS-PAGE and then transferred onto polyvinylidene fluoride (PVDF) membranes (Millipore, Bedford, MA, USA). The membranes were blocked in 5% bovine serum albumin (BSA) in 1 × TBST for 1 h, followed by incubation with rabbit polyclonal anti-GOLPH3 (Abcam, ab236296), anti-STIP1 (Cell signaling Technology, Danvers, MA, USA; #2080), anti-c-Myc (Cell signaling Technology, #5605), anti-cyclin D1 (Cell signaling Technology, #2978), or mouse monoclonal anti-GAPDH (glyceraldehyde-3-phosphate dehydrogenase; Cell signaling Technology, #5174) at 4 °C overnight. After washing the membranes were incubated with horseradish peroxidase conjugated goat anti-rabbit immunoglobulin G (IgG) secondary antibody (Cell signaling Technology, #14708, 1:10000) for 1 h and then the immunoreactive proteins were visualized using an enhanced chemiluminescence (ECL) kit (Millipore, Bedford, MA, USA). GAPDH was used as an internal control.

### Immunohistochemistry (IHC) and Immunofluorescence (IF)

Paraffin-embedded sections were deparaffinized, rehydrated and prepared for antigen retrieval as described previously (17). After blocking with 10% goat serum, sections were incubated with mouse polyclonal anti-GOLPH3 (Abcam, ab69171), or anti-STIP1 antibodies (Abcam, ab126753), or anti-Cyclin D1, or anti-Ki67 (Cell signaling Technology, #2586), or anti-Bim (Cell signaling Technology, #2933) at 4°C overnight. Then, biotin-labeled secondary antibodies were added for 1 h at room temperature.

A score criteria was assigned to evaluate the percentage of positively stained PDAC tissues, as previously reported (17). Briefly, the level of GOLPH3 or STIP1 staining was based on the proportion of positively stained tumor cells (area of staining) and the intensity of staining. The percentage of stained tumor cells was classified into 4 grades (0, no positive tumor cells; 1, <10% positive tumor cells; 2, 10–35% positive tumor cells; 3, 35–70% positive tumor cells; 4, >70% positive tumor cells); while the cell staining intensity was classified into 4 levels (0–3+) (0, no staining; 1, weak staining or light yellow; 2, moderate staining or yellow brown; 3, strong staining or brown color). The two values were multiplied to obtain the IHC score (staining index, SI), giving SI values of 0, 1, 2, 3, 4, 6, 8, 9, 12, as previously described (25). The optimal cutoff values were SI ≥ 6 to define tumors with high GOLPH3 or STIP1 expression, and SI ≤ 4 to define tumors with low GOLPH3 expression. All the IHC scores were repeated three times using a double-blind method.

Cells for the IF assay were grown on glass coverslips which were fixed for 20 min on ice in 1 × phosphate buffered saline (PBS; pH 7.4) containing 4% paraformaldehyde, incubated in permeabilization solution (0.5% Triton-X 100, 20 mM HEPES, 50 mM NaCl, 3 mM MgCl_2_, and 300 mM Sucrose) for 10 min, followed by a second permeabilization for 30 min at room temperature after washing in 1 × PBS. The coverslips were then blocked in 3% goat serum with 0.1% BSA in 1 × PBS followed by incubation with primary antibodies (overnight at 4°C) and secondary antibodies (1 h at room temperature). The primary antibodies were the same as those described above. The secondary antibodies were fluorescein-conjugated goat anti-rabbit/mouse IgG (DyLight549, LK-GAM4881, Liankebio, Hangzhou, China).

### Cell Culture and siRNA Transfection Targeting *GOLPH3* or *STIP1*

Human PDAC cell lines PANC1 and BXPC3 were purchased from the Shanghai Cell Bank (Shanghai, China). PANC1 cells were cultured in DMEM (Gibco) with 10% FBS, 50 U/mL pf penicillin, and 50 μg/mL of streptomycin (Thermo Fisher Scientific, Waltham, MA, USA), while BXPC3 cells were cultured in Roswell Park Memorial Institute (RPMI)-1640 medium (Gibco). All cells were cultured in a sterile incubator to maintain at 37°C with 5% CO_2_. The short interfering RNAs (siRNAs) targeting *GOLPH3* and *STIP1* and control siRNAs were purchased from Thermo Fisher Scientific. The siRNA sequences for *GOLPH3* were 5′-GGACCGCGAGGGTTACACATCATTT-3′ (siRNA-1) and 5′-GCATTGAGAGGAAGGTTACAACTAG-3′ (siRNA-2) and 5′-GGAATGACTGTATATCATCTGGATT-3′ (siRNA-3). The siRNA sequences for *STIP1* were 5′-GCAAGACTGTCGACCTAAA-3′ (siRNA-1) and 5′-CGATGAAGGACTACACCAA-3′ (siRNA-2). The Silencer siRNA product as siRNA negative control was purchased by Thermo Fisher Scientific (#12935200). Cells were transfected with either *GOLPH3* or *STIP1* or control siRNAs using Lipofectamine® RNAiMAX (Thermo) according to the manufacturer's instructions.

### Real-Time Quantitative-Telomere Repeat Amplification Protocol (Q-TRAP) for Relative Telomerase Activity

Cells (5 × 10^6^) were lysed on ice for 30 min in NETN buffer [1 M Tris-HCl pH 8.0, 1 mM EDTA, 100 mM NaCl, 0.5% NP-40, 1 mM DTT, and proteinase/RNase inhibitor cocktail (Sigma)], diluted to 1 × 10^4^ cells/μL, and then centrifugated at 12,000 rpm for 10 min at 4°C. The supernatant was diluted 2 to 5-fold before being used for PCR. Each 25 μL Q-TRAP reaction contained 2 μL of the eluted proteins, 100 ng each of the TS primer (5′-AATCCGTCGAGCAGAGTT-3′) and ACX primer (5′-GCGCGGCTTACCCTTACCCTTACCCTAACC-3′), and 1 mM EGTA, in SYBR Green PCR Master Mix (Applied Biosystems, Foster City, CA, USA). The reaction mixtures were incubated at 30°C for 30 min and then PCR-amplified (40 cycles of 95°C for 15 s and 60°C for 60 s) using a LightCycler 480 SYBR Green I Master (Roche, Boston, MA, USA).

### RNA Extraction and Quantitative Real-Time Reverse Transcription PCR (qRT-PCR)

Total RNA from cells or tissues was extracted using TRIPURE ISOLATION REAGENT (Roche) according to the manufacturer's protocol. The RNA was treated with RNase-free DNase in advance, and the 2 μg of RNA from each specimen was reverse transcribed to cDNA. Quantitative real-time PCR (qPCR) of the cDNA was conducted using the Roche 480 system (Roche) using a LightCycler 480 SYBR Green I Master Mix. The following primer sequences were used: *GOLPH3*, sense (5′-CTCCAGAAACGGTCCAGAAC-3′),antisense (5′-CCACCAGGTTTTTAGCTAATCG-3′); *MYC*(*c-Myc*), sense (5′-CACCAGCAGCGACTCTGA-3′), antisense (5′-GATCCAGACTCTGACCTTTTGC-3′); *CCND1* (*cyclin D1*), sense (5′-GTGCTGCGAAGTGGAAACC-3′), antisense (5′-ATCCAGGTGGCGACGATCT-3′); *hTERT*, sense (5′-CACCAGCAGCGACTCTGA-3′), antisense (5′-ATCCAGACTCTGACCTTTTGC-3′); *GAPDH*, sense (5′-CTGACTTCAACAGCGACACC-3′), antisense (5′-TGCTGTAGCCAAATTCGTTG-3′). Denaturation was performed at 95°C for 30 s followed by 35 annealing cycles of 60°C for 20 s. *GAPDH* expression was used as an internal reference when conducting the data analysis.

### Cell Cycle Analysis, CCK8 Cell Proliferation Assay, Ethynyl deoxyuridine (EdU) Incorporation Assay, and Colony Formation Assays

Cells were harvested, washed with PBS, and fixed in 75% ethanol. RNase A (20 μg/mL) and propidium iodide staining solution (50 μg/mL) were added to the cells and incubated for 30 min. BD Biosciences FACS Calibur Flow Cytometry (BD Biosciences, Franklin Lakes, NJ, USA) was then used for cell-cycle analysis.

To assess cell growth and viability, a Cell Counting Kit-8 (CCK8, Dojindo, Japan) was used. Briefly, the protocol was as follows: PANC1 and BXPC3 cells (5 × 10^3^ cells per well) were cultured in a 96-well plate. After 12 h (time for the cells to attach to the plate surface), 10 μL of CCK8 solution was added to each well and incubated for 4 h. The cell viability was then determined by measuring the absorbance of the converted dye at 450 nm. EdU (5-ethynyl-2′-deoxyuridine) incorporation assay was carried out using the Cell-Light TM EdU imaging detecting kit according to the manufacturer's instructions (RiboBio, Guangzhou, China). The EdU is a thymidine analog whose incorporation can be used to label cells undergoing DNA replication. Cells (5 × 10^4^) were seeded into each well of the 96-well plate. After the cells attached to the plate surface, culture medium with 5 μM EdU was added to cells and incubated for 2 h. The cells were fixed using 4% formaldehyde at room temperature for 30 min and then treated with Apollo reaction cocktail for 30 min. Finally, DNA was stained using Hoechst 33342 (Thermo Fisher Scientific).

For the colony formation assay, at 48 h after transfection, 500 cells were seeded in a 6-well plate and cultured for 12 days. The colonies were stained with 1% crystal violet for 2 min after fixation with 10% formaldehyde for 15 min. Each treatment was repeated three times.

### Expression of shGOLPH3 in BXPC3 Cells and Xenografts

BXPC3 cells (5 × 10^6^) stably transformed with psi-LVRU6GP-shGOLPH3 (expressing a small hairpin RNA (shRNA) targeting *GOLPH3*) or psi-Vector obtained from FulenGen (Guangzhou, China) were suspended in 150 μL PBS and injected subcutaneously into the right axilla of female BALB/c nude mice, respectively (4 weeks old, *n* = 5 per group). shRNA sequence for *GOLPH3* was 5′-GAGAGGAAGGTTACAACTA-3′ and Lentiviral particles for scramble control product as negative control was purchased from FlulenGen (# LPP-CSHCTR001-LVRU6GP-100). Ten mice were purchased from Charles River Laboratories (Beijing, China) and randomly divided into two groups for the construction of a subcutaneous tumorigenesis model. The sizes of the tumors were measured every 3 days using calipers and their volumes were calculated according the formula: length × width^2^/2. Mice were sacrificed by cervical dislocation on day 25 and the xenografts were removed, fixed with 4% paraformaldehyde, embedded in paraffin, and sectioned. Sections were stained with hematoxylin and eosin (H&E), and with GOLPH3, STIP1, Ki67, Cyclin D1, and Bim antibodies to assess cell proliferation in the tumor samples. The animal study was reviewed and approved by the Institutional Animal Care and Use Ethics Committee of First Affiliated Hospital of Sun Yat-sen University (Ethical code number: [2015]51). All animal experiments performed in accordance with animal protocols.

### Statistical Analysis

All statistical analyses were performed using the Statistical Package for the Social Sciences (SPSS) 22.0 software (IBM Corp., Armonk, NY, USA). The associations between GOLPH3 and STIP1 proteins expression was analyzed by Chi-square test and correlation analysis. Statistical analysis was performed using Student's *t*-test for comparison between two groups, and one-way ANOVA was used for comparison between multiple groups. Counting data were expressed as number or rate. Data were presented as means ± standard deviation (SD) and each experiment was repeated at least three times. *P* < 0.05 was considered statistically significant.

## Results

### Screening and Validation of GOLPH3's Interaction With STIP1

**Figure 1:**
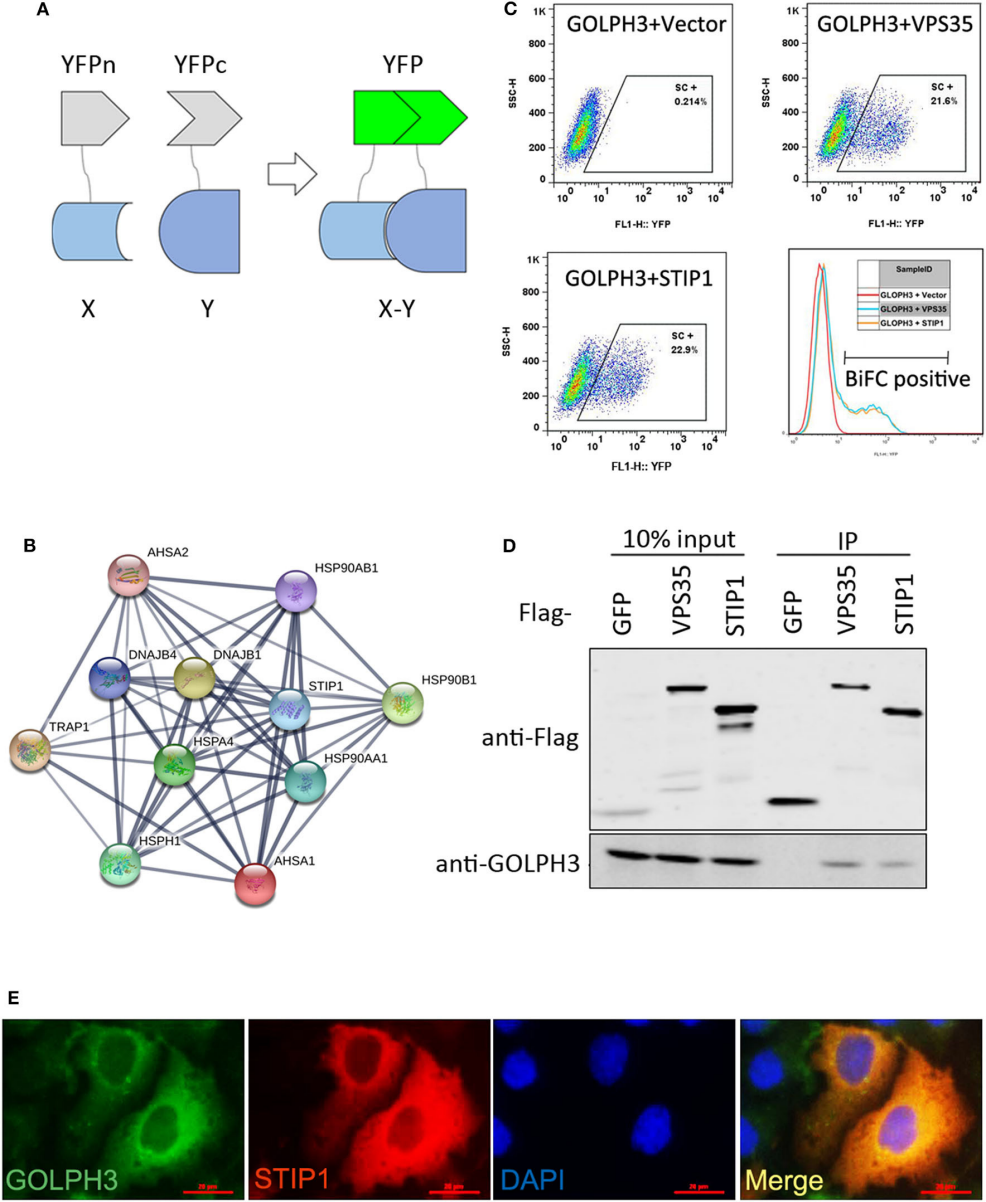
GOLPH3 interacts with STIP1. **(A)** A schematic diagram of the BiFC screening assay. Non-fluorescent subfragments of a fluorescent protein YFPn and YFPc were fused to X and Y, respectively. If X and Y interact, the two fragments could associate and refold, allowing fluorescence to occur. **(B)** BiFC screening for GOLPH3 interacting proteins and a map of the BiFC results for protein interaction networks constructed using STRING. **(C)** FACS analysis in HTC75 cells co-expressing BiFC variants of GOLPH3, VPS35, and STIP1; and quantitative analysis of YFP fluorescence intensity from the panel represented as a histogram. **(D)** The Co-IP result showing the interaction between GOLPH3 and VPS35 and STIP1. **(E)** Immunofluorescence showing GOLPH3 and STIP1 co-expressed in PDAC cells.

To explore the interaction of proteins with GOLPH3 in living cells, we developed a genome-wide screening strategy based on the BiFC assay. The BiFC system is based on the reconstitution of two non-fluorescent fragments of a fluorescent protein (26). The two halves of YFP (YFPn and YFPc), respectively tagged to proteins X and Y through a ten-amino acid (AA) peptide linker, are brought together for co-folding when X and Y interact. The schematic diagram of the assay used in this study is shown in Figure 1A. To better understand the protein interaction network of GOLPH3, we carried out a BiFC screen using a YFPn-GOLPH3 stable expression cell line and a YFPc-tagged ORFeome library that contained 18,000 human ORFs. We first generated a human HTC75 cell line stably expressing YFPn-GOLPH3 by retrovirus infection and G418 selection. The YFPc-tagged human ORFeome library was pooled together and then used to produce retroviruses to infect the GOLPH3-YFPn HTC75 expressing cell line, at a multiplicity of infection (MOI) of about 0.5. After puromycin selection, YFP positive cell populations were isolated using flow cytometry. The cDNAs from the ORFeome library enriched in the YFP positive cells were PCR amplified and sequenced using next-generation sequencing. After bioinformatic analysis, a high-resolution map of the GOLPH3 interacting network based on our BiFC screening data was established using website software STRING 11 (http://string-db.org/). Among these candidates, we identified several proteins that bound significantly to GOLPH3 (Figure 1B). Among them, STIP1 ranked at the top of this list and emerged as a robust GOLPH3 interacting protein. STIP1, also known as heat shock protein (HSP) 70/HSP90-organizing protein, is a 62.6 kDa protein that is capable of interacting with HSPs and plays multiple roles in cell cycle regulation and signal transduction (27–29). Previous investigations have shown that STIP-1 overexpression is associated with tumor aggression and poor prognosis in various types of cancer (30–32). VPS35, which has been shown to interact with GOLPH3, was used as a positive-binding control. Our pair-wise BiFC assay results suggested that GOLPH3 interacted with VPS35 and STIP1 (Figure 1C). To further confirm the interaction between GOLPH3 and STIP1, immunoprecipitation results indicated that endogenous GOLPH3 not only associated with Flag-tagged VPS35, but also with STIP1 (Figure 1D). Immunofluorescence assays using a bright yellow fluorescence signal in the cytoplasm of PDAC cells suggested that GOLPH3 and STIP1 were co-localized in the cytoplasm (Figure 1E). These data confirmed the interaction between GOLPH3 and STIP1.

### Overexpression of GOLPH3 Is Related to the Level of STIP1 in PDAC

**Figure 2:**
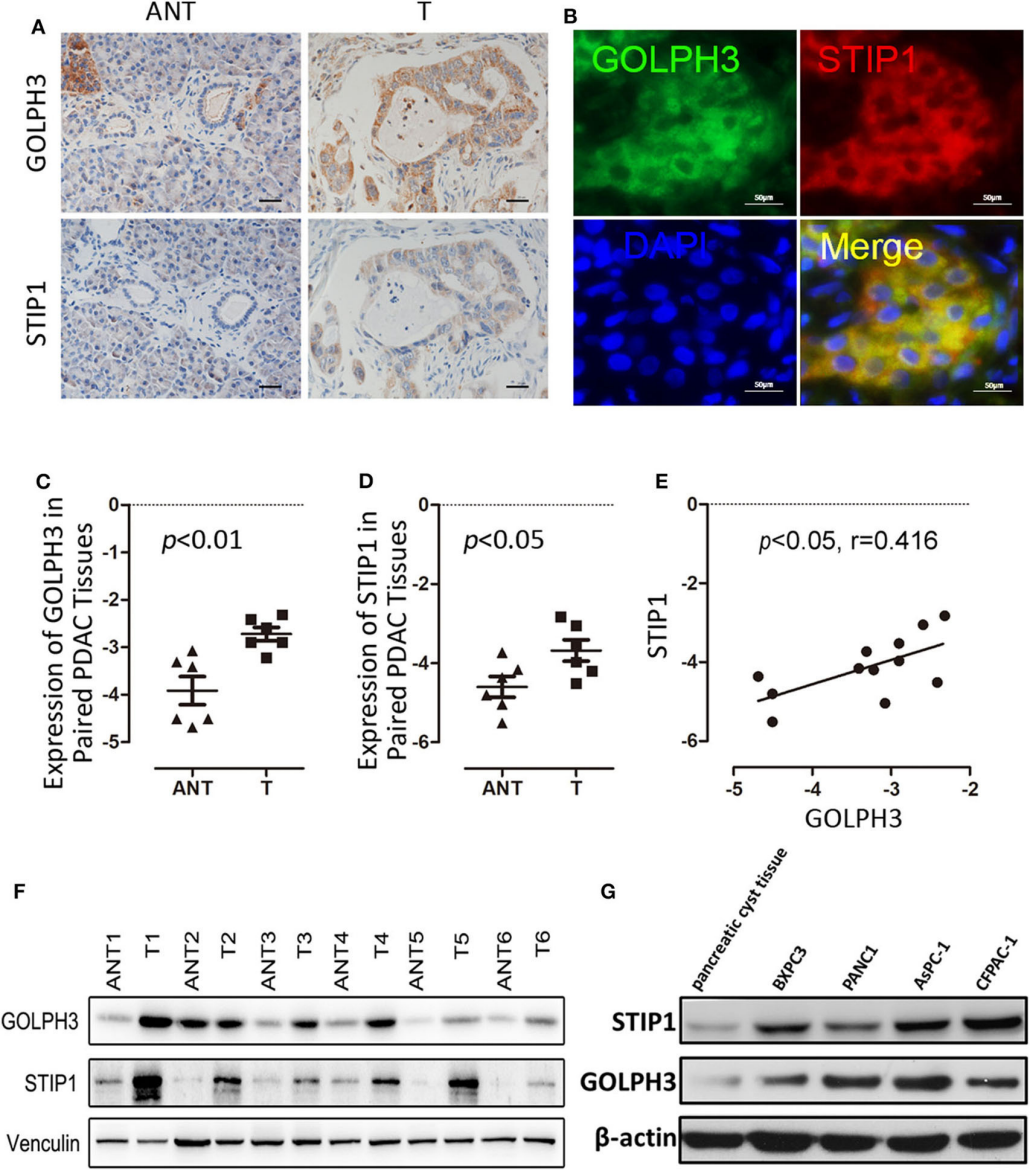
GOLPH3 correlates positively with STIP1 in PDAC tissues. **(A)** Immunohistochemical staining for GOLPH3 and STIP1 in PDAC tissues (T) and paired normal pancreatic tissues (ANT). **(B)** Co-expression of GOLPH3 and STIP1 in PDAC tissues as assessed using immunofluorescence. **(C)** Expression of *GOLPH3* analyzed using qRT-PCR in paired normal and PDAC tissues. **(D)** *STIP1* levels were measured in paired PDAC tissues and adjacent normal tissues using qRT-PCR. The data were normalized to *GAPDH* levels as a control. **(E)** Correlation of GOLPH3 and STIP1 expression in PDAC tissues. **(F)** GOLPH3 and STIP1 protein levels were measured in paired PDAC tissues using western blotting. **(G)** The expression of GOLPH3 and STIP1 in different PDAC cell lines compared with that in pancreatic cyst tissue as a control.

Using IHC and IF assays, we evaluated the expression of GOLPH3 and STIP1 in six paired tumor and adjacent non-cancerous tissue specimens from patients with PDAC. We found that the STIP1 level was increased in tumor tissue (T) compared with that in adjacent non-cancerous tissue (ANT), and correlated positively with the GOLPH3 level (Figure 2A). Notably, the subcellular localization of GOLPH3 and STIP1 was observed to be mainly in the cytoplasm of the cancer cells (Figure 2B). Furthermore, qRT-PCR assays showed that GOLPH3 and STIP1 expression levels were significantly upregulated in PDAC tissues compared with those in normal tissues (Figures 2C,D). Moreover, overexpression of GOLPH3 correlated positively with STIP1 expression in PDAC tissues (Figure 2E). In addition, GOLPH3 and STIP1 protein levels were dramatically upregulated in PDAC tissues compared with those in their matched para-cancer counterparts (Figure 2F). Similarly, the expression of GOLPH3 and STIP1 were higher in PDAC cell lines than those in the control groups (Figure 2G). In our previous work, we revealed that 72.5% (79/109) of PDAC cases showed high GOLPH3 expression, whereas 27.5% (30/109) showed low GOLPH3 expression (17). Therefore, immunohistochemical staining for STIP1 was also performed on those 109 FFPE PDAC tissue samples. The results showed that 45% (49/109) of the PDAC cases displayed high STIP1 expression, whereas 55% (60/109) displayed low STIP1 expression (Table 1). There were 49 cases with high STIP1 expression among the 79 patients showing high GOLPH3 expression, and STIP1 expression was found related to GOLPH3 levels (*r* = 0.5569, *p* < 0.05), as shown in Table 1. Our results confirmed the positive correlation between GOLPH3 and STIP1. Taken together, these results suggested that GOLPH3 interacts with STIP and both proteins might be involved in the development of PDAC.

**Table 1:** Correlation assay between GOLPH3 and STIP1.

		**GOLPH3**		**Total**	**Correlation**
		**High**	**Low**		
STIP1	High	49 (45%)	0 (0)	49 (45%)	*p* < 0.05 *r* = 0.5569
	Low	30 (27.5%)	30 (27.5%)	60 (55%)	
Total		79 (72.5%)	30 (27.5%)	109 (100%)	

### Knockdown of *GOLPH3* or *STIP1* downregulates hTERT/Cyclin D1 by c-Myc in PDAC Cells

**Figure 3:**
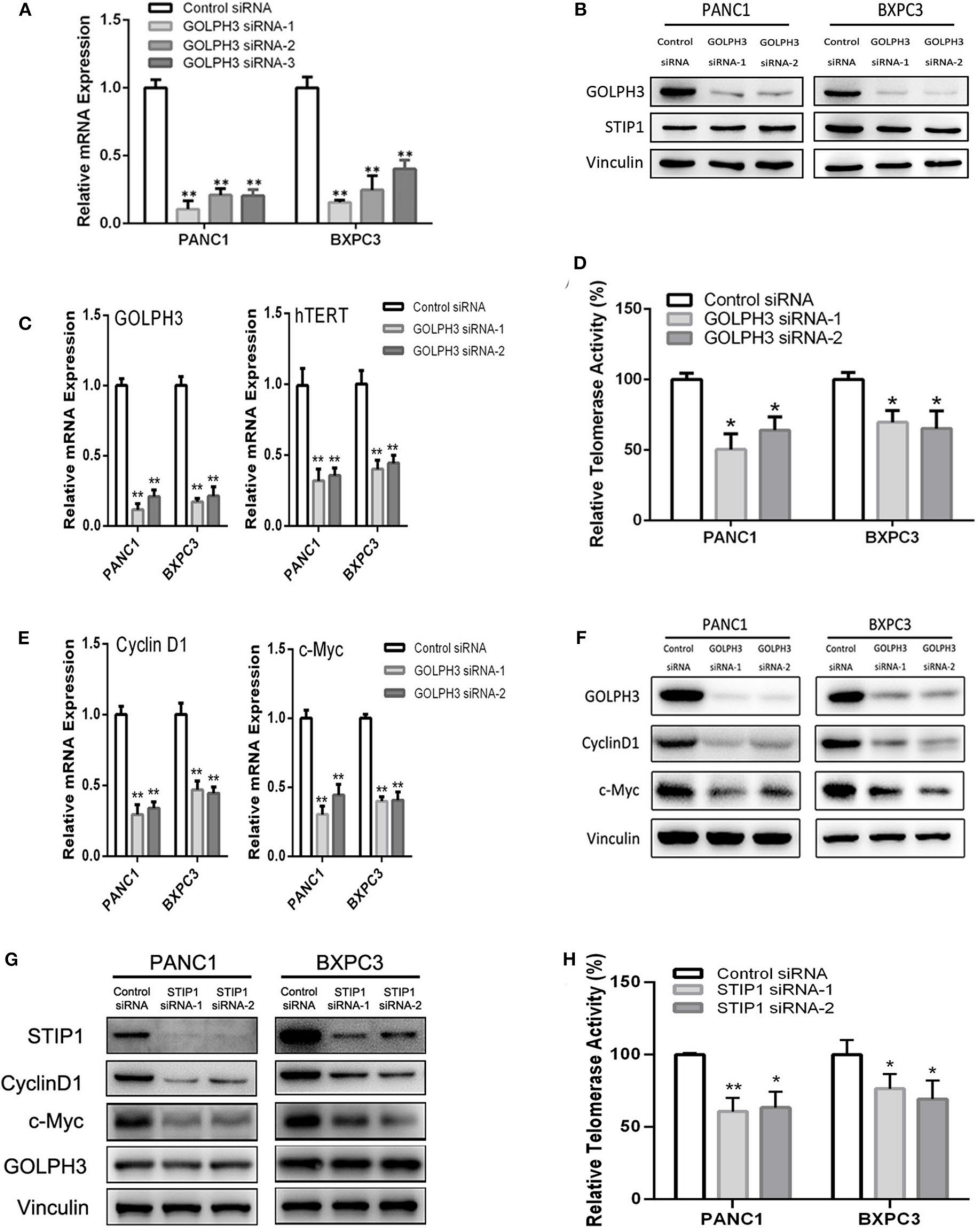
The interaction of GOLPH3 and STIP1 regulates telomerase activity in PDAC cells. **(A)** *GOLPH3* siRNA downregulates GOLPH3 expression mRNA levels in PANC1 and BXPC3 cells. **(B)** *GOLPH3* siRNA downregulates GOLPH3 but not STIP1 levels in PANC1 and BXPC3 cells. **(C)** Expression of GOLPH3 and hTERT transfected with control siRNA and *GOLPH3* siRNA-1/2 were assessed using qRT-PCR. **(D)** The relative activity of telomerase in the control and *GOLPH3* knockdown of PDAC cells detected using q-TRAP. **(E,F)** Expression of c-Myc and Cyclin D1 transfected with the control siRNA and *GOLPH3* siRNA-1/2 were assessed using qRT-PCR and western blotting (E mRNA and F Protein). **(G)** Expression of Cyclin D1, c-Myc, and GOLPH3 transfected with the control siRNA and *STIP1* siRNA-1/2, assessed using qRT-PCR. **(H)** The relative activity of telomerase in the control and *STIP1* knockdown PDAC cells detected using q-TRAP. *p < 0.05 and **p < 0.01.

The BiFC assay provided evidence that GOLPH3 interacts with STIP1 and heat shock protein group including HSP90 (Figure 1C). As a 60kDa protein that belonging to one of the co-chaperones families, STIP1 reversibly binds to the protein chaperones HSP90, which regulates c-Myc expression in cancerous cells (33, 34). c-Myc activates *hTERT* transcription *via* E-box binding, resulting in direct regulation of telomerase activity (35). Therefore, we explored the possibility that the GOLPH3/STIP1 complex regulates hTERT and subsequently activates telomerase activity. We designed siRNAs targeting *GOLPH3* or *STIP1* to knock down their expression in PANC1 and BXPC3 cells, respectively. The qRT-PCR results showed that the relative expression levels of *GOLPH3* mRNA were effectively decreased in the siRNA-transfected group (Figure 3A). Therefore, siRNA-1 and siRNA-2 were used in all subsequent experiments. It was noted that there was no change in STIP1 level when knocking down *GOLPH3* compared with those in the control cells (Figure 3B). The results showed that c-Myc levels were dramatically suppressed by transfecting PDAC cells with *GOLPH3* or *STIP1*-specific siRNAs (Figures 3F,G). Importantly, we found that the levels of *hTERT* and the relative telomerase activity were drastically reduced in the cells transfected with the *GOLPH3* or *STIP1* siRNA compared with those in the control cells (Figures 3C,D,H). Many studies have shown that inhibition of hTERT expression arrests the cell cycle of cancer cells and cell cycle-related signaling, including that involving cyclin D1 (36, 37). Consequently, we found that the cyclin D1 level was significantly decreased in *GOLPH3* knockdown cells compared with those in the control cells (Figures 3E,F). Moreover, knockdown of *STIP1* using siRNAs markedly decreased the protein levels of cyclin D1 in PANC1 and BXPC3 cells, whereas there was no change in the expression of GOLPH3 (Figure 3G). These results suggested that GOLPH3's interaction with STIP1 is a key factor for c-Myc binding to the *hTERT* promoter, and the subsequent regulation of downstream cyclin D1 in PDAC cells.

### Effect of *GOLPH3* Knockdown on PDAC Cell Proliferation

**Figure 4:**
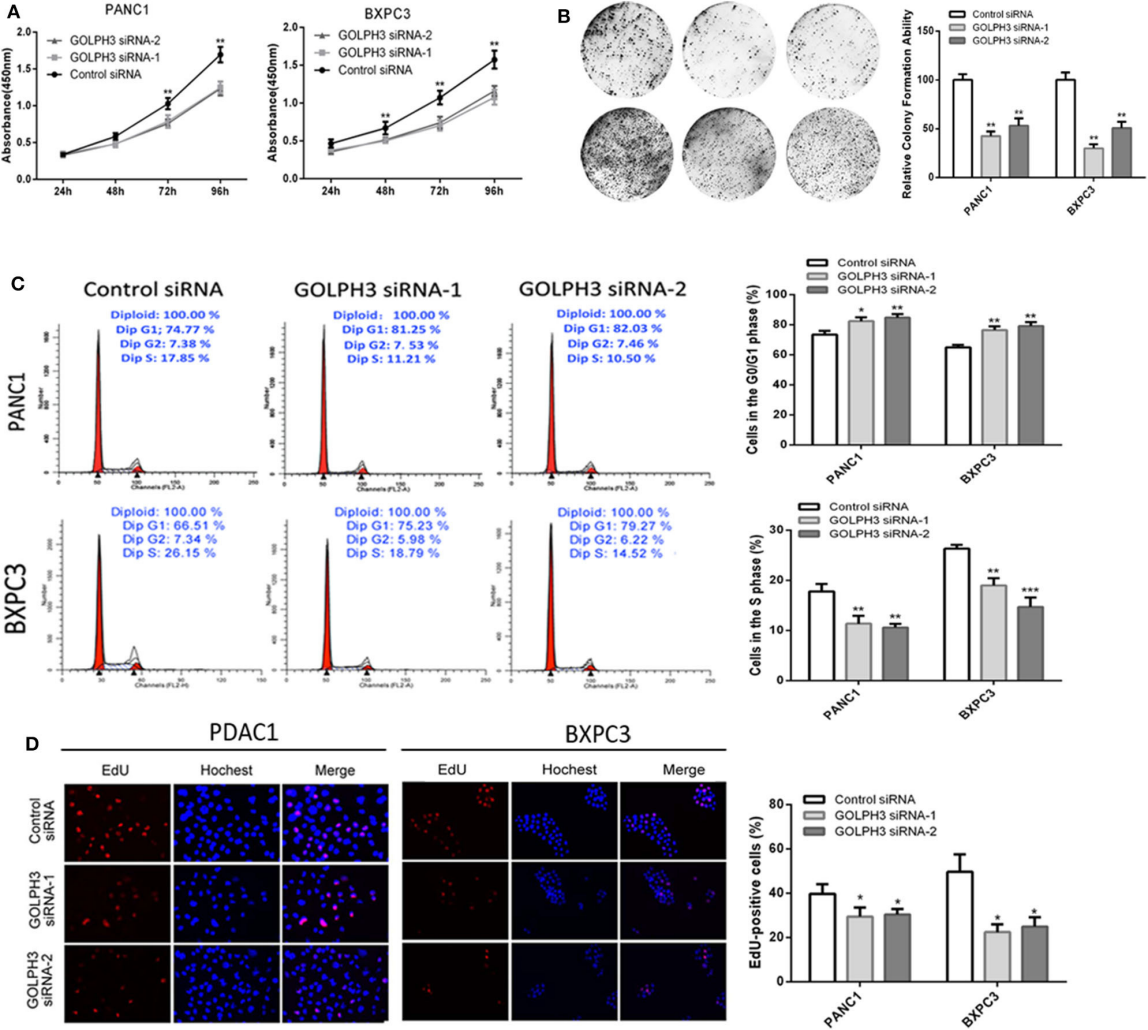
Knockdown of *GOLPH3* inhibits cell proliferation. **(A)** *GOLPH3* siRNA inhibited proliferation of PDAC cells as detected using CCK-8 assays at different time points. **(B)** *GOLPH3* siRNA inhibited colony formation in pancreatic cancer cells. **(C)** Cell cycle assay to determine the role of GOLPH3 in cell cycle progression after downregulating *GOLPH3*. **(D)** EdU staining assay to determine the effect of GOLPH3 downregulation on cell proliferation. *p < 0.05, **p < 0.01, and ***p < 0.001.

Increasing evidence suggests that hTERT affects tumor cell proliferation (36); therefore, we examined whether the GOLPH3 expression level and relative telomerase activity affected the proliferation in PDAC cells. The CCK8 assay showed that the cell proliferation rate was significantly reduced in a time-dependent manner in PANC1 and BXPC3 cells transfected with *GOLPH3* siRNA compared with that of the control cells (Figure 4A). The result was further confirmed by the colony formation assay, which showed a clear reduction in the number of colonies formed by both cell lines transfected with the *GOLPH3* siRNA (Figure 4B). These data indicated that suppression of GOLPH3 expression is sufficient to inhibit PDAC cell proliferation. Li et al. demonstrated that hTERT regulates G0/G1-S phase transition to influence cell proliferation (36). Therefore, we further measured the GOLPH3-mediated cell cycle using flow cytometry and EdU incorporation. Silencing of *GOLPH3* increased the percentage of cells in the G0/G1 phase and decreased the percentage of S phase cells (Figure 4C). In parallel, knockdown of GOLPH3 significantly reduced the percentage of EdU-incorporating cells (Figure 4D). These results suggested that GOLPH3 has a marked effect on cell proliferation by contributing to the G0/G1–S-phase transition in PDAC cells.

### Effect of *STIP1* Knockdown on PDAC Cell Proliferation

**Figure 5:**
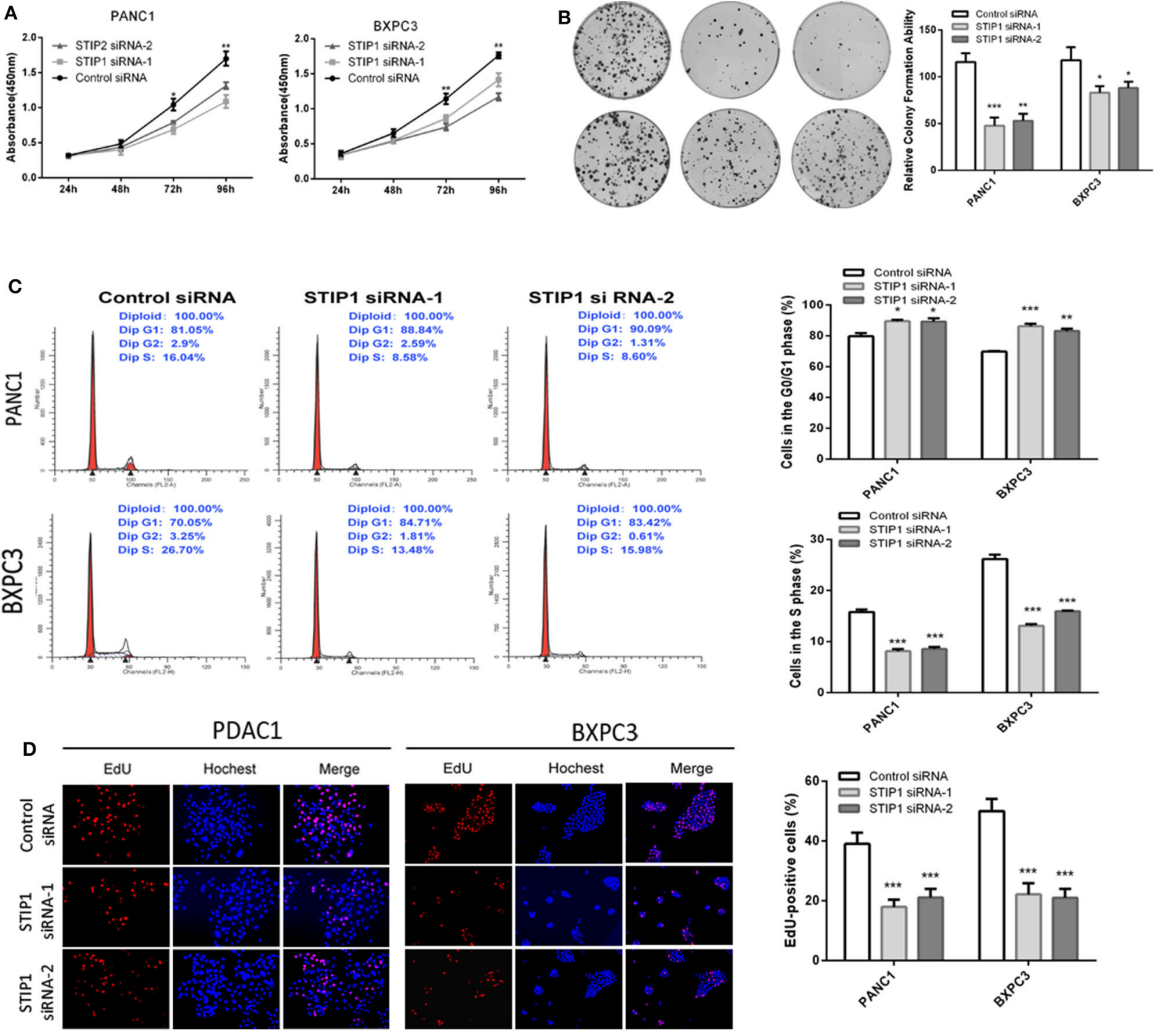
Knockdown of *STIP1* inhibits cell proliferation. **(A)** *STIP1* siRNA inhibited proliferation of PDAC cells as detected using CCK-8 assays at different time points. **(B)** *STIP1* siRNA inhibited colony formation in pancreatic cancer cells. **(C)** Cell cycle assay to determine the role of STIP1 in cell cycle progression after downregulating *STIP1*. **(D)** EdU staining assay to determine the effect of STIP1 downregulation on cell proliferation. *p < 0.05, **p < 0.01, and ***p < 0.001.

Besides GOLPH3, we also detected the effect of *STIP1* on cell proliferation in PDAC cells. The CCK8 assay showed that when knockdown of *STIP1*, cell proliferation was inhibited in both PANC-1 and BXPC3 cells (Figure 5A). We performed colony formation assay to further validate the proliferation ability of STIP1 in PDAC cells. The results confirmed that when STIP1 was knockdown, number of cell colonies were significantly less than that of control cells (Figure 5B). We further examined the effect of STIP1 on cell cycle progression in PDAC cells. The results showed that cell cycle was arrested at the G1 phages when PDAC cells transfected with siRNAs (Figure 5C). Moreover, the EdU incorporation assay showed that STIP1 suppression inhibited cell proliferation compared to control groups (Figure 5D). These results illustrate that STIP1 can promote cell proliferation and accelerate cell cycle in PDAC.

### Effect of *GOLPH3* Knockdown on PDAC Tumorigenicity and Relative Telomerase Activity *in vivo*

**Figure 6:**
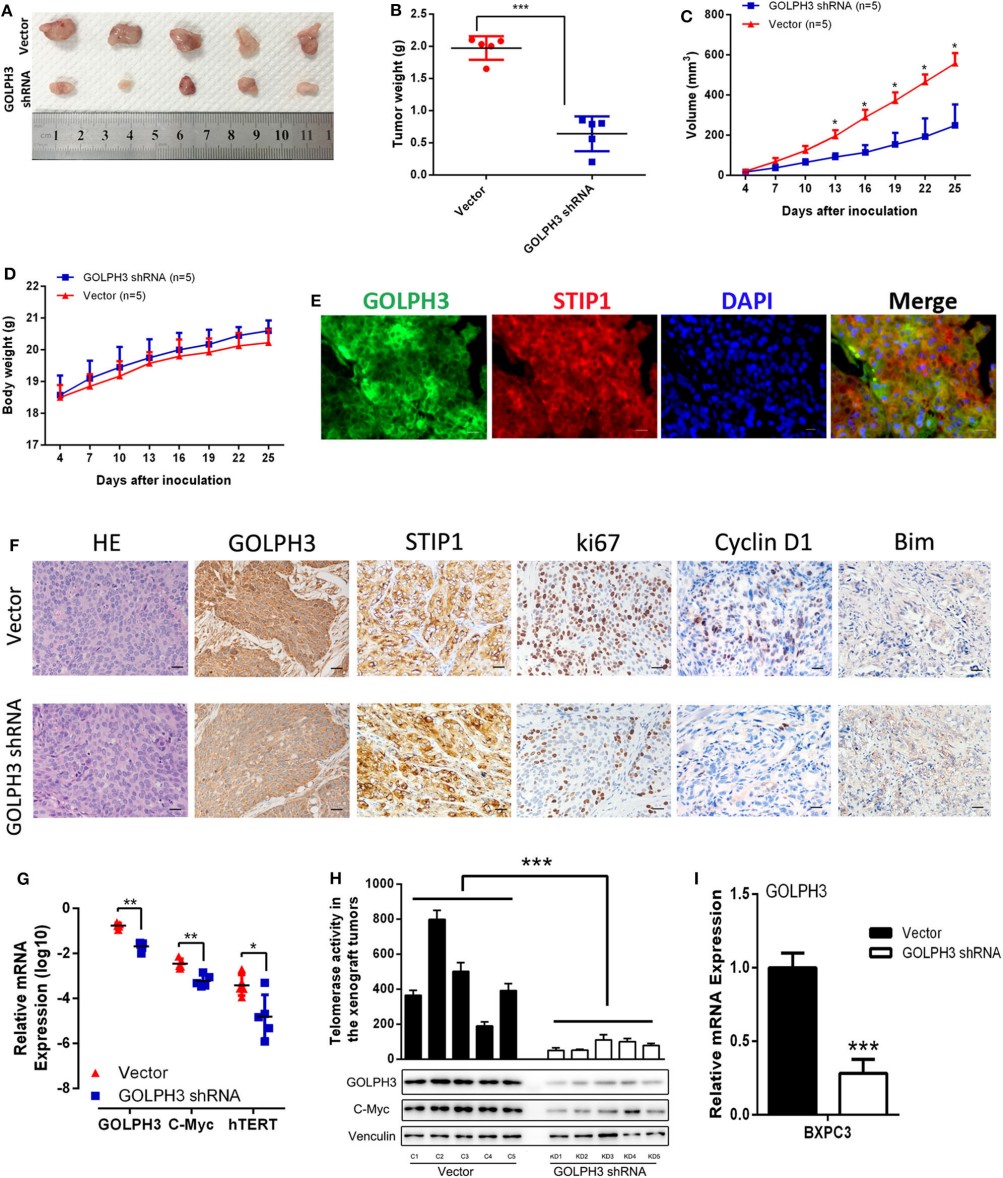
GOLPH3 promotes the tumorigenicity of PDAC cells *in vivo*. **(A)** Xenograft model in nude mice; representative image of tumors from all mice in each group. **(B)** Dots graphs represent the weight of the BXPC3 cells xenograft tumor after sacrificing mice. **(C)** Tumor volume growth curves by the indicated cells. Growth of subcutaneous xenografts was weakened by downregulation of GOLPH3 in nude mice. **(D)** Time courses of animal weight. **(E)** IF assay to detect GOLPH3 and STIP1 localization in subcutaneous xenografts. **(F)** HE staining and IHC of GOLPH3, STIP1, ki67, Cyclin D1, and Bim in subcutaneous xenografts in nude mice. **(G)** qRT-PCR analysis of *GOLPH3, c-Myc*, and *hTERT* mRNA expression *in vivo*. **(H)** The relative activity of telomerase in paired PDAC tissues in the xenograft tumors (upper panel) and GOLPH3, c-Myc, and hTERT expression by western blotting analysis (lower panel). **(I)** The relative expression of GOLPH3 in GOLPH3 shRNA group and vector group. *p < 0.05, **p < 0.01, and ***p < 0.001.

At first, we confirmed GOLPH3 expression was significantly inhibited in GOLPH3 shRNA group than that in control group (Figure 6I). Our *in vitro* data showed that *GOLPH3* knockdown significantly reduced cell proliferation. To confirm the role of GOLPH3 in regulating the growth of PDAC tumor *in vivo*, a PDAC xenograft nude mouse model was constructed using the PDAC cell line BXPC3. Tumor growth was observed for 25 days. The tumor volume of *GOLPH3* shRNA group was significantly reduced compared with those in the vector group (*p* < 0.05; Figures 6A,B). As shown in Figure 6C, the xenografts grew significantly slower in the *GOLPH3* shRNA group compared with that in the vector group. Meanwhile, body weight of the nude mice did not markedly change during these experiments (Figure 6D). Furthermore, double immunofluorescent staining revealed co-localization of GOLH3 and STIP1 proteins in the cytoplasm in xenografts tissues, which indicated there might be an interaction between GOLPH3 and STIP1 (Figure 6E). Moreover, GOLPH3, STIP1, Ki67, Cyclin D1, and Bim protein levels were analyzed within the xenografts using IHC. The positive expression of GOLPH3, Ki67, and Cyclin D1 proteins in the *GOLPH3* shRNA group were significantly lower than that in the vector group, while the expression of proapoptotic protein Bim was opposite. It also should be noted that STIP1 expression were not significantly changed in the both groups, which indicated there might be an bimolecular interaction between GOLPH3 and STIP1 but not one was upstream/downstream of another to activate the signaling pathway (Figure 6F). These results indicate knocking down GOLPH3 can inhibit cell proliferation and accelerate cell apoptosis compared to control group, which is consistent with experimental data *in vitro*. Furthermore, the levels of c-Myc and hTERT were downregulated in the *GOLPH3* shRNA group compared with those in the vector group (Figures 6G,H). In addition, the relative telomerase activity of the tumors was also decreased in the *GOLPH3* shRNA group compared with that in the vector group (Figure 6H). Collectively, the above *in vivo* findings confirmed the *in vitro* results, and indicated that GOLPH3 promotes tumor growth by upregulating hTERT levels and telomerase activity through c-Myc.

## Discussion

The mortality rate of PDAC is high because diagnosis usually occurs at an advanced stage. Except for surgical resection, there have been no significant improvements regarding the treatment of PDAC over recent decades. Therefore, there is an urgent need to understand the molecular oncogenesis underlying PDAC to provide new therapeutic strategies. Accumulating evidence shows that oncoprotein GOLPH3 participates in the development of a variety of cancer types (19). Previously, we reported that GOLPH3 overexpression was significantly associated with poor clinical outcome in PDAC (17). Our data showed that GOLPH3 was highly expressed in PDAC tissues. This was consistent with the results obtained from other studies in several types of cancer, which demonstrated that *GOLPH3* might play an oncogenic role in tumorigenesis (14, 15, 18, 38, 39). Consequently, it would be interesting to determine how the molecular functions of GOLPH3 are linked to the regulation of PDAC progression. Protein-protein interactions play an important role in tumor progression. Previous studies have verified that GOLPH3 interacts with different proteins that are involved in the development of various tumors. Taft et al. reported an interaction of GOLPH3 with myosin-18A that maintains a tensile force in the Golgi. Scott et al. proposed that GOLPH3 might function with VPS35 to affect cell malignant transformation *via* enhancing the activity of mTOR (11, 40). The interaction between GOLPH3and VPS35 plays an important role in regulating mTOR signaling and promoting uncontrolled cell proliferation. Although the contribution of GOLPH3 to tumor growth and metastasis is well-supported; molecular pathways linking GOLPH3 to its oncogenic signaling are yet to be mapped in PDAC. In this report, we focused on novel proteins that interact with GOLPH3, which might play important roles in the tumor growth and development of PDAC. Therefore, we investigated PPIs using the BiFC screening assay. In BiFC, interacting proteins are expressed as fusion proteins with fragments of YFP: Amino-YFP (YFPn) or carboxyl-YFP (YFPc). Individual fusion proteins do not possess intrinsic fluorescence unless an interaction occurs between proteins leading to factional YFP assembly (41). After screening 18,000 proteins, we identified a novel GOLPH3-binding protein, STIP1. Binding and colocalization of GOLPH3 and STIP1 were verified using Co-IP and immunofluorescence co-localization. The results also showed that the expression levels of GOLPH3 and STIP1 were higher in tumor tissues than in adjacent non-cancerous tissues. In addition, our results further indicated that GOLPH3 expression correlated positively with STIP1 expression in PDAC tissues, and their co-localization in tumor cells implied their interaction. Our findings revealed that STIP1 is a binding partner of GOLPH3. In addition, a more in-depth investigation is needed to screen small molecules as anti-tumor drugs to inhibit the intracellular interaction of GOLPH3 and STIP1 in PDAC.

STIP1 has been reported to be overexpressed in PDAC cell lines and malignant tissue samples from patients with PDAC (27). STIP1 is proposed as a co-chaperone of HSP90 that regulates c-Myc expression in cancerous cells (34). It is reported that c-Myc interacts with an E-box in the *hTERT* promoter resulting in expression of hTERT and telomerase (35). Telomerase is an RNA-dependent DNA polymerase that is involved in synthesizing telomeric repeats to repair telomeres. Telomerase is normally inactivated in most somatic cells, while it contributes to tumorigenesis during telomere maintenance (35). The catalytic subunit hTERT has the most important role in telomerase's function. Kumari et al. demonstrated that telomerase activity and hTERT levels are upregulated in PDAC, which could be predictors of poor outcome in patients with PDAC (42). Numerous studies have suggested that telomerase activity and hTERT levels correlate with c-Myc activation during the early stage of carcinogenesis (35). Moreover, c-Myc is an important transcription factor that promotes the expression of cyclin D1 during cell proliferation and tumor development (36). Park et al. has disclosed that hTERT is significantly related to cyclin D1 expression (43). Based on these findings, we hypothesized that GOLPH3 associated with STIP1 might regulate hTERT and telomerase activity *via* c-Myc, and then regulate cyclin D1 to promote the division and proliferation of tumor cells. As expected, hTERT expression and telomerase activity as well as c-Myc and cyclin D1 levels, were downregulated in GOLPH3 or STIP1-silenced PDAC cells, which in turn promoted PDAC cell proliferation. Moreover, GOLPH3 knockdown seemed to have no effect on the expression of STIP1 and vice versa. Our results suggested that GOLPH3 has a bimolecular interaction with STIP1 to activate c-Myc/hTERT/cyclin D1 signaling pathway to promote PDAC cell proliferation. More evidence needs to prove the critical bimolecular interaction between GOLPH3 and STIP1 but not one was upstream/downstream of another in the signaling axis in our further research. It has been reported that AKT is activated by phosphoinositide-dependent kinases 1 (PDK1), and then signal transduction occurs through downstream effectors such as mTOR to increase cancer cell proliferation (44). Interestingly, we found that GOLPH3 enhanced PDK1, mTOR, and AKT phosphorylation in the PDK1/mTOR/AKT signal pathway (data was not shown), which indicated crosstalk between the c-Myc/hTERT/cyclin D1 and PDK/mTOR/AKT signaling pathways, and could explain how the GOLPH3-STIP1 interaction induced tumor cell proliferation.

Previous studies have shown that cyclin D1 is the most important signaling molecule related to cell proliferation and could regulate the “restriction point” of the G0/G1 phase. The G0/G1 phase is the most important switch for cell proliferation, after which cells can enter S phase and complete the division cycle. Therefore, the cell division competence of a cell population is reflected by the number of cells at G0/G1 phase (45). Li et al. found that hTERT could regulate the G0/G1 to S phase transition (36). In the present study, *GOLPH3* silencing inhibited cell proliferation by inhibiting the G0/G1–S-phase transition in PDAC cells. Similarly, colony-formation and Edu assays also revealed that knockdown of *GOLPH3* inhibited the cells' proliferative activity. Thus, our data provides new insights into the association between the GOLPH3/STIP1 complex and cell proliferation. Additionally, shRNA-transfected BXPC3 cells were transplanted into nude mice to investigate the effect of GOLPH3 on tumor growth *in vivo*. The results revealed that depletion of GOLPH3 significantly suppressed tumor growth in the nude mice. Consistent with our finding, Xue et al. demonstrated that *GOLPH3* knockdown inhibited the tumorigenicity of cancer cells in a xenograft mouse model (25). Further analysis of the grafted tumors verified that GOLPH3 downregulation was associated with a reduction in c-Myc and hTERT expression, as well as reduced telomerase activity, in the GOLPH3-suppressed group compared with that in the vector group. Huang et al. reported that in a nude mouse model, STIP1 could promote lung metastasis in gastric cancer (46). Therefore, whether the GOLPH3-STIP1interaction affects metastasis in PDAC remains to be determined in a future study.

In summary, this study used BiFC to identify novel the GOLPH3-binding protein STIP1 and provided evidence that GOLPH3 and STIP1 are overexpressed in PDAC tissues and cell lines. We demonstrated that the GOLPH3-STIP1 interaction regulates hTERT expression *via* c-Myc, and then affects cell cycle-related signaling including cyclin D1 leading to the promotion of PDAC proliferation and tumor growth. These findings could provide new insights to develop therapeutic targets to treat PDAC. In the future, large scale screening might lead to the identification of small molecule chemicals that inhibit the GOLPH3-STIP1 interaction.

## Data Availability Statement

The raw data supporting the conclusions of this article will be made available by the authors, without undue reservation.

## Ethics Statement

The studies involving human participants were reviewed and approved by the Institutional Ethical Review Boards of the First Affiliated Hospital of Sun Yat-sen University (Ethical code number: 201515). The patients/participants provided their written informed consent to participate in this study. This animal study was reviewed and approved by the Institutional Animal Care and Use Ethics Committee of First Affiliated Hospital of Sun Yat-sen University (Ethical code number: [2015]51).

## Author Contributions

WL and ZS designed the research study. KW and SJ performed the majority of the experiments and data analysis. BP and ZL contributed to specimen preparation. AH, SZ, and YG assisted with the *in vivo* experiments. MH and WM wrote the manuscript. All authors approved the final version of the manuscript.

## Conflict of Interest

The authors declare that the research was conducted in the absence of any commercial or financial relationships that could be construed as a potential conflict of interest.
